# Extreme value modelling of SARS-CoV-2 community transmission using discrete generalized Pareto distributions

**DOI:** 10.1098/rsos.220977

**Published:** 2023-03-08

**Authors:** Abdelaati Daouia, Gilles Stupfler, Antoine Usseglio-Carleve

**Affiliations:** ^1^ Toulouse School of Economics, University of Toulouse Capitole, Toulouse, France; ^2^ University of Angers, CNRS, LAREMA, SFR MATHSTIC, 49000 Angers, France; ^3^ Laboratoire de Mathématiques d’Avignon UPR 2151, Avignon Université 84000 Avignon, France

**Keywords:** COVID-19, superspreading, cluster size, secondary cases, extreme value theory, discrete extremes

## Abstract

Superspreading has been suggested to be a major driver of overall transmission in the case of SARS-CoV-2. It is, therefore, important to statistically investigate the tail features of superspreading events (SSEs) to better understand virus propagation and control. Our extreme value analysis of different sources of secondary case data indicates that case numbers of SSEs associated with SARS-CoV-2 may be fat-tailed, although substantially less so than predicted recently in the literature, but also less important relative to SSEs associated with SARS-CoV. The results caution against pooling data from both coronaviruses. This could provide policy- and decision-makers with a more reliable assessment of the tail exposure to SARS-CoV-2 contamination. Going further, we consider the broader problem of large community transmission. We study the tail behaviour of SARS-CoV-2 cluster cases documented both in official reports and in the media. Our results suggest that the observed cluster sizes have been fat-tailed in the vast majority of surveyed countries. We also give estimates and confidence intervals of the extreme potential risk for those countries. A key component of our methodology is up-to-date discrete generalized Pareto models which allow for maximum likelihood-based inference of data with a high degree of discreteness.

## 1. Introduction

Superspreading events (SSEs) have been recognized as a significant source of disease transmission for respiratory coronaviruses such as SARS-CoV and SARS-CoV-2 [1,2]. SSEs may be defined as outbreaks in which a given individual (the index case) infects a number of people (secondary cases) well above a certain measure, such as the average or median number of infections. The number of secondary cases resulting directly from an index case can be viewed as a random variable, say *Z*, defining the so-called offspring distribution. For both coronaviruses, events having triggered more than six secondary cases have been suggested to constitute SSEs [3]. Data on such SSEs that were curated and reported in [3] in the early stages of the COVID-19 pandemic is necessarily scarce: it consists mainly of 15 SSEs associated with SARS-CoV and 45 SSEs associated with SARS-CoV-2, each represented by a number of secondary cases *Z*_*i*_ resulting from a single given index case in Europe, Asia or North America. The natural framework for the analysis of SSEs, and more generally of atypical observations far away from the mean, is extreme value theory. Following this framework, it was argued in [3] that SSEs are fat-tailed, although this was done by pooling the 60 available SSEs from SARS-CoV and SARS-CoV-2. A careful investigation of these SARS-CoV and SARS-CoV-2 datasets reveals that the two largest observations in the pooled data are SARS-CoV SSEs; given the small sample size, one may wonder whether the reported estimate of tail heaviness is representative of the tail behaviour of SARS-CoV-2 SSEs.

This constitutes the motivation for this work, whose overarching goals are to show how to conduct a statistically rigorous extreme value analysis of community transmission parameters, and to carry out such an analysis in the example of SARS-CoV-2. By focusing directly on the raw SARS-CoV-2 data considered in [3], we provide evidence of a lighter upper tail for SSEs with significantly less tail exposure than predicted in their study. We arrive at the same conclusion by making use of a more recent and much larger publicly available surveillance and contact-tracing database containing the number of secondary cases *Z*_*i*_ for 88 527 index cases in the Indian states of Andhra Pradesh and Tamil Nadu [4]. We also analyse two other South Korean contact-tracing datasets, one collected in the first half of 2020 [3], the other during the summer of 2021, when the Delta variant of SARS-CoV-2 was responsible for the majority of positive cases [5]. The fat-tailedness of the secondary cases distribution is found to be rather clear in the 2021 sample of data, while the analysis of the 2020 data is less conclusive. In all these samples of data, we find point estimates of the extreme value index suggesting that the secondary cases distribution has a finite third moment, which stands in contrast with the earlier finding of Wong & Collins [3] of a distribution with an infinite variance.

In addition to that, we consider the broader problem of large community transmission, as it represents the other fundamental source of pandemic risk. Large infection clusters, along with SSEs, have been argued to play an important role in the transmission of SARS-CoV-2 [2]. In a similar spirit to Adam *et al.* [2], we define a cluster of SARS-CoV-2 cases in our analysis as a local outbreak involving a minimum of two cases, including confirmed close contacts with epidemiological linkage over a limited period of time. We consider two databases constructed from government reports [6–9] and media sources [10], comprising 15 samples of SARS-CoV-2 cluster sizes recorded in 11 countries and four US states. Our results show that 13 of these 15 countries and states have fat-tailed cluster size distributions, thus facilitating the process of inferring their risk category in terms of large community transmission. This allows us to better understand the drivers of superspreading and cluster formation in the ongoing COVID-19 pandemic. The recent theory of discrete extremes [11–14] is our basic tool to address the highly discrete nature of SARS-CoV-2 secondary transmission data and cluster sizes. Its use constitutes our main statistical contribution to the study of the transmission of the SARS-CoV-2 virus. As we illustrate throughout the paper, estimating and inferring the extreme value index and extreme percentiles of the underlying discrete distributions with this methodology is much easier and more accurate than with classical extreme value methods such as the Hill and generalized Pareto maximum likelihood estimators, which heavily rely on the continuous data assumption.

The structure of the paper is as follows. We first describe the methods employed throughout our study, including the discrete generalized Pareto distribution fitted to exceedances over a high threshold by means of the maximum likelihood estimator. We then analyse our datasets, first on SARS-CoV-2 secondary case numbers and then on cluster sizes, using these methods. The final section gathers and contrasts these findings and concludes with additional comments about the scope, limitations and robustness of our results, as well as ideas for further work.

## 2. Methods

We use several methods from extreme value theory, which constitutes the correct mathematical framework for the analysis of high observations from a random phenomenon [15]. We are particularly interested in methods that can describe so-called fat-tailed random variables, which infrequently but regularly generate very high values and therefore appear to be relevant in the analysis of SARS-CoV-2 transmission. A random variable *X* is fat-tailed if and only if its distribution function P(X≤x) can be, for large *x*, expressed as $P(X\leq x)=1-x^{-1/\xi }\ell (x)$, where ℓ satisfies ℓ(*tx*)/ℓ(*t*) → 1 as *t* → ∞ for any positive real number *x*. Informally, the tail behaviour of *X* is controlled by the extreme value index *ξ* > 0, which must be estimated to get a precise understanding of tail heaviness. A standard estimator in this context is the Hill estimator [16]. For a dataset $Z_{1},\ldots ,Z_{n}$, the Hill estimator at threshold *u* is defined as

$${\hat{\xi }}_{u}^{H}=\frac{1}{\sum_{i=1}^{n}1_{\left\{Z_{i}>u\right\}}}\sum_{i=1}^{n}\log\left(\frac{Z_{i}}{u}\right)1_{\left\{Z_{i}>u\right\}}.$$

It is of course crucial, before using the Hill estimator, to ascertain whether the distribution of the data points indeed has a heavy tail. A common diagnostic method is the mean excess plot, which estimates the values of the mean excess function E(u)=E[Z−u|Z>u] as function of *u*. A natural estimate of *E*(*u*) is given, for each threshold *u*, by its empirical counterpart

$$\hat{E}(u)=\frac{\sum_{i=1}^{n}Z_{i}1_{\left\{Z_{i}>u\right\}}}{\sum_{i=1}^{n}1_{\left\{Z_{i}>u\right\}}}-u.$$

A fat-tailed distribution will typically have mean excess plots exhibiting a linear upward drift for large values of *u*, whose slope is a consistent estimate of *ξ*/(1 − *ξ*) when *ξ* < 1, see for example section 1.2.2, pp. 14–19 and p. 152 in [17]. In the case *ξ* ≥ 1, Theorem 3.4 and Remark 3.5 in [18] show that the mean excess plot converges in a suitable sense to a random curve, which in the log–log scale is a straight line with slope 1/*ξ* and random intercept term constructed upon a stable random variable with index 1/*ξ*.

It has, however, been observed in the extreme value literature [18] that the mean excess function very often exhibits a nonlinear behaviour at the right end of the mean excess plot, due to very high variability of the estimate of *E*(*u*) when *u* is close to the highest *Z*_*i*_. As a consequence, good statistical practice recommends to confirm a diagnostic of a heavy tail using other extreme value tools. One such general approach, which does not presuppose that the data are fat-tailed, consists in using the generalized Pareto maximum likelihood estimator applied to the excesses *Z*_*i*_ − *u*. Recall that the generalized Pareto distribution, with shape parameter *ξ* and scale parameter *σ*, has probability density function

$$h_{\xi ,\sigma }(x)=\frac{1}{\sigma }{\left(1+\xi \frac{x}{\sigma }\right)}^{-1/\xi -1}1_{\left\{x>0,\;1+\xi x/\sigma >0\right\}}.$$

The generalized Pareto maximum likelihood estimator is then defined as, according to section 5.3.2 in [17] and [19]

$$\begin{array}{rl} \left({\hat{\xi }}_{u}^{GP},{\hat{\sigma }}_{u}^{GP}\right) & =\underset{\xi >-1,\;\sigma >0}{\arg\min}\sum_{i=1}^{n}\log h_{\xi ,\sigma }(Z_{i}-u)\\ & =\underset{\xi >-1,\;\sigma >0}{\arg\min}\sum_{i=1}^{n}\left[-\log\sigma -\left(\frac{1}{\xi }+1\right)\log\left(1+\xi \frac{Z_{i}-u}{\sigma }\right)\right]1_{\left\{Z_{i}>u,\;1+\xi (Z_{i}-u)/\sigma >0\right\}}. \end{array}$$

The generalized Pareto maximum likelihood estimators are valid even when the underlying distribution is not fat-tailed, which has made them very popular in the natural sciences [20].

**Figure 1.**
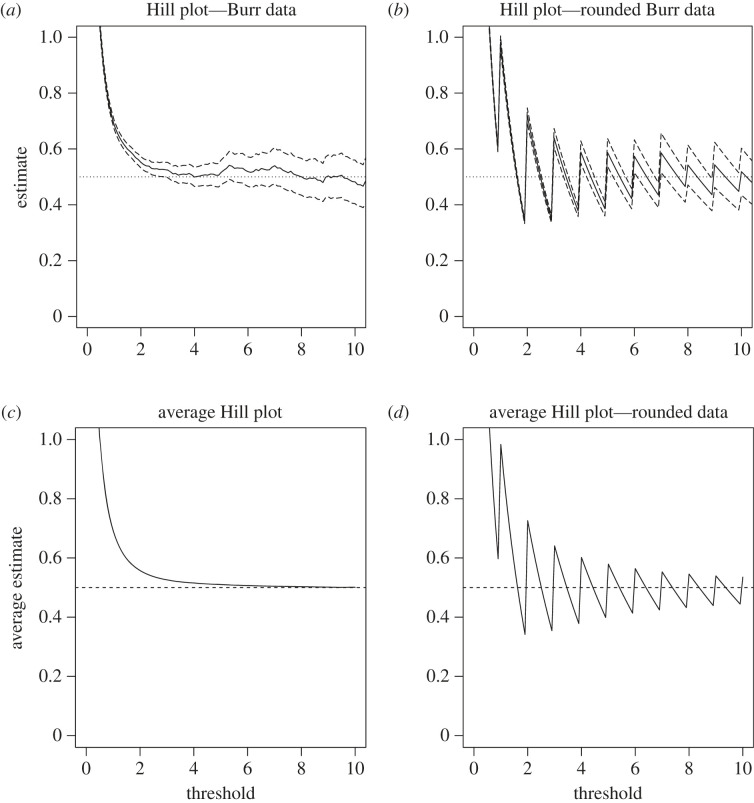
(*a,b*) Hill plots (solid lines) and corresponding 90% Gaussian asymptotic confidence intervals (dashed lines) as functions of the threshold value *u*, for *n* = 10 000 simulated data points *Z*_*i*_ from the Burr distribution with probability density function *f*(*x*) = *ξ*^−1^ *x*^−*ρ*/*ξ*−1^ (1 + *x*^−*ρ*/*ξ*^)^1/*ρ*−1^ (for *x* > 0) with *ξ* = 1/2 and *ρ* = −1 in (*a,c*), and for the data $\left\lceil Z_{i}\right\rceil$ (i.e. the smallest integer larger than or equal to *Z*_*i*_) in (*b,d*). (*c,d*) Averaged Hill plots when this experiment is repeated *N* = 1000 times.

However, both the Hill and generalized Pareto estimators of *ξ* suffer from jagged sample paths when the data points *Z*_*i*_ feature a substantial number of ties, that is, they come from a distribution with a high degree of discreteness. This behaviour makes it extremely difficult to choose an accurate estimate of *ξ*, which renders the two methods highly unsatisfactory. The essential reason behind this phenomenon is that both estimators are built under the—generally incorrect—assumption that the data points come from a pure (generalized) Pareto distribution, which is continuous, and as such, they cannot be expected to handle a substantial degree of discreteness. We exemplify this phenomenon in figure 1*a,b*: notice the stark difference in stability and smoothness of sample paths between a plot of the Hill estimator as a function of the threshold value (henceforth referred to as a Hill plot) for continuous data *Z*_*i*_ and its counterpart for data rounded to the nearest integer up. Crucially in applied set-ups, the asymptotic Gaussian confidence intervals constructed by approximating the distribution of $\sqrt{nP(X>u_{n})}({\hat{\xi }}_{u_{n}}^{H}-\xi )$ by a Gaussian distribution with expectation 0 and variance *ξ*^2^, which is valid when *u*_*n*_ → ∞ satisfies reasonable conditions [21], are highly unstable when the data features a large number of ties, thus making inference using the Hill estimator unadvisable. The bottom panels of this figure (figure 1*c,d*) further show the impact of these data ties: the Hill estimator for discrete data tends to be strongly biased and much more so than the Hill estimator for continuous data.

An alternative option properly taking the discreteness of the data into account is to employ discrete models to construct an estimator of the extreme value index. This was pursued by Shimura [11] and Hitz *et al.* [13], which used so-called D-GPD (for discrete generalized Pareto distribution) models, first employed by Prieto *et al.* [12] to model road accidents and more recently by Ranjbar *et al*. [14] to model hospital congestion. The D-GPD, whose probability mass function is

$$p_{\xi ,\sigma }(x)={\left(1+\xi \frac{x}{\sigma }\right)}^{-1/\xi }-{\left(1+\xi \frac{x+1}{\sigma }\right)}^{-1/\xi }\;\text{ for }x=0,1,2,\ldots \text{ with }p_{\xi ,\sigma }(x)>0,$$

for *ξ* ≥ 0 or *ξ* < 0 and *σ*/*ξ* a negative integer, has been shown to outperform the continuous GPD when there are a large number of tied observations: see the simulated Poisson and discrete inverse-Gamma examples in section 3.1 of Hitz *et al.* [13], which, respectively, show that the GPD provides poor model fits and poor tail estimates when the data are highly discrete, while the D-GPD distribution performs well. Its closed-form survival and probability mass functions allow for an exact likelihood-based inference constructed upon the maximum likelihood estimators

$$({\hat{\xi }}_{u},{\hat{\sigma }}_{u})=\underset{\xi >-1,\;\sigma >0}{\arg\min}\sum_{i=1}^{n}\log p_{\xi ,\sigma }(Z_{i}-u).$$

When *ξ* = 0, the convention we adopt is that (1 + *ξz*)^−1/*ξ*^ = exp ( − *z*), for any z∈R. These maximum likelihood estimators of the extreme value index *ξ* and scale parameter *σ* of the D-GPD model are readily obtained through the R maximization routine optim. Using the classical theory of maximum likelihood estimators, confidence intervals for *ξ* may be derived from ${\hat{\xi }}_{u}$ by estimating the total Fisher information matrix *I*(*ξ*, *σ*) using a finite difference method and then deducing the following 100α% confidence interval for *ξ*

$$\left[{\hat{\xi }}_{u}+\sqrt{{\left(\hat{I}{\left(\xi ,\sigma \right)}^{-1}\right)}_{1,1}}\;\Phi^{-1}\left(\frac{1-\alpha }{2}\right),\;{\hat{\xi }}_{u}+\sqrt{{\left(\hat{I}{\left(\xi ,\sigma \right)}^{-1}\right)}_{1,1}}\;\Phi^{-1}\left(\frac{1+\alpha }{2}\right)\right],$$

where Φ denotes the standard normal distribution function and $\Phi^{-1}$ its inverse (quantile function). Modelling *Z* − *u* conditional on *Z* ≥ *u* by a D-GPD distribution with parameter estimates $\left({\hat{\xi }}_{u},{\hat{\sigma }}_{u}\right)$ suggests the following estimate of the 100*α*th percentile of *Z* adapted from ([12], formula (5), p. 41):

$${\hat{q}}_{\alpha }=\left\lceil \frac{{\hat{\sigma }}_{u}}{{\hat{\xi }}_{u}}\left({\left(\frac{n(1-\alpha )}{\sum_{i=1}^{n}1_{\left\{Z_{i}\geq u\right\}}}\right)}^{-{\hat{\xi }}_{u}}-1\right)+u-1\right\rceil ,$$

for *α* ∈ (0, 1) large enough. Here, ⌈⋅⌉ denotes the ceiling function, that is, ⌈x⌉ denotes the smallest integer larger than or equal to *x*. Estimating this quantile by plugging in the aforementioned estimates of *ξ* and *σ* makes it possible to infer extreme quantile levels and therefore get precise information on the tail behaviour of a distribution with a large degree of discreteness. For each of the extreme value estimators we have introduced (Hill estimator, GPD and D-GPD maximum likelihood estimators), a common practice for selecting a suitable pointwise estimate of *ξ*, colloquially referred to as ‘eyeballing’, is to pick out a sufficiently high threshold *u* corresponding to a stable region of the plot [15]. We shall indeed also adopt this practice and will clearly indicate selected thresholds or threshold regions in our analyses.

For comparison purposes, we will contrast the resulting extreme quantile estimates with those provided by the (conditioned) negative binomial distribution. Recall that the probability mass function of the negative binomial distribution (with parameters *r* > 0 and *p* ∈ (0, 1)) conditional on *Z* > *u*, is given by

$$P_{\;p,r,u}(Z=k)=\frac{(\Gamma (k+r)/\left(k!\;\Gamma (r)\right))p^{r}{\left(1-p\right)}^{k}}{1-\sum_{i=0}^{u}(\Gamma (i+r)/\left(i!\;\Gamma (r)\right))p^{r}{\left(1-p\right)}^{i}},\;\text{ for all }k>u.$$

Here Γ denotes Euler’s Gamma function. With a dataset $z_{1},\ldots ,z_{n}$, the parameter estimators are, therefore, obtained as the maximum log-likelihood solution

$$\underset{\left(p,r)\in (0,1)\times (0,\infty \right)}{\arg\max}\sum_{i=1}^{n}\log P_{\;p,r,u}(Z=z_{i}).$$

Ever since the seminal work of Lloyd-Smith *et al.* [1], the negative binomial distribution has been widely used to describe the number of secondary cases resulting from an index case of SARS-CoV. As suggested in [3,22], this model has exponentially decreasing probability mass functions and thus cannot be expected to accurately represent tail heaviness in SARS-CoV-2 transmission data. We provide below further evidence for this claim, and for the suitability of D-GPD maximum likelihood estimates in the context of discrete data, through several datasets gathering numbers of SARS-CoV-2 secondary cases and cluster sizes in different settings.

## 3. Data and results

### 3.1. Analysis of secondary case data

Our first two datasets were reported in [3]. They consist of 15 SSEs associated with SARS-CoV (Dataset S1) and 45 SSEs associated with SARS-CoV-2 (Dataset S2), each resulting in more than six secondary cases, along with month of occurrence and location of the superspreading event, and its setting. We refer to Wong & Collins [3] for further details about the construction of these datasets. Pooling the 15 SSEs associated with SARS-CoV and 45 SSEs associated with SARS-CoV-2 into a single sample and making use of a generalized Pareto approximation, Wong & Collins [3] have suggested that the distribution of the number of secondary cases *Z* belongs to the Fréchet maximum domain of attraction [23], that is, the set of Pareto-type distributions, with extreme value index *ξ* between 0.5 and 1 (the estimate provided in [3], fig. 1E is $\hat{\xi }\approx 0.6$). The index *ξ* tunes the tail heaviness of the distribution, with higher positive values indicating a heavier upper tail: moments of order higher than or equal to 1/*ξ* do not exist. An estimate of *ξ* around 0.6 means that the second moment of *Z* does not exist, reflecting the outsized contribution of SSEs to overall transmission. Most importantly perhaps, these findings on the tail heaviness of *Z* invalidate the conventional assumption that *Z* follows a negative binomial distribution for either coronavirus, whereas this assumption was widely adopted in the literature on disease transmission ever since the influential work [1] on SARS-CoV, and it is still widely employed for SARS-CoV-2 (e.g. [5,24,25]).

**Figure 2.**
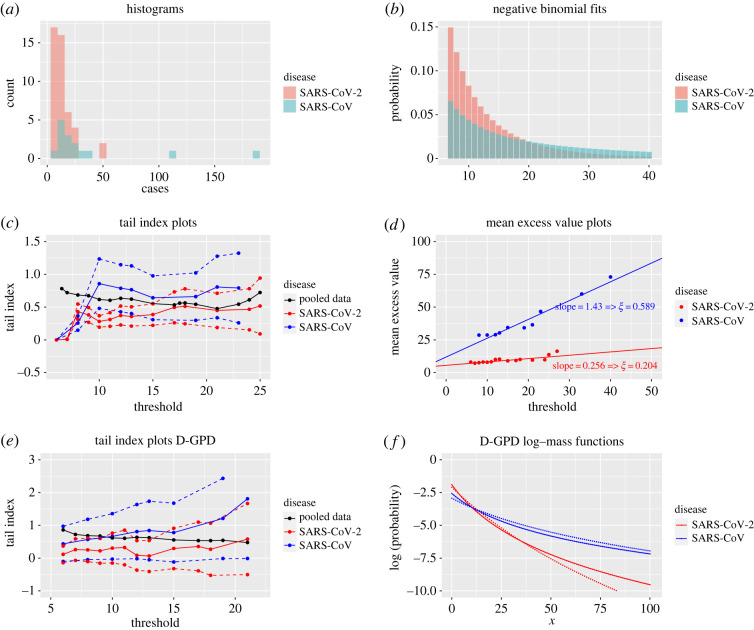
Secondary case data from Wong & Collins [3] (Datasets S1 and S2). (*a*) Histogram of the number of secondary cases for SARS-CoV (blue, *n* = 15) and SARS-CoV-2 (red, *n* = 45) SSEs. (*b*) Fitted probability mass function, conditional on *Z* > 6, of the negative binomial distribution for SARS-CoV (blue) and SARS-CoV-2 (red) SSEs. (*c*) Hill estimates of *ξ* for SSEs associated with SARS-CoV (solid blue), SARS-CoV-2 (solid red), and the pooled data (solid black), obtained from the exceedance values *Z*_*i*_ − *u* given *Z*_*i*_ ≥ *u*, as function of the threshold *u*, along with the resulting 90% confidence intervals for SARS-CoV (dashed blue) and SARS-CoV-2 (dashed red) SSEs. (*d*) Mean excess plots of SARS-CoV (blue) and SARS-CoV-2 (red) SSEs, quantified by the average of the exceedances *Z*_*i*_ − *u* given *Z*_*i*_ ≥ *u*, as function of *u*. (*e*) Discrete GPD maximum likelihood estimates of *ξ* for SARS-CoV (solid blue) and SARS-CoV-2 (solid red) SSEs, calculated from the exceedances *Z*_*i*_ − *u* given *Z*_*i*_ ≥ *u*, as function of *u*, along with their corresponding 90% confidence intervals (dashed lines), and the Hill plot produced by combining SARS-CoV and SARS-CoV-2 SSEs (black line). (*f*) Logarithm of the probability mass functions $P_{\sigma ,\xi }(X=x)$ of the D-GPD fits to the exceedance values *Z*_*i*_ − *u* given *Z*_*i*_ ≥ *u*, for the thresholds *u* = 6 (dotted lines) and *u* = 10 (solid lines), for SARS-CoV (blue) and SARS-CoV-2 (red).

Based on our statistical analysis of these datasets, summarized in figure 2, one may, however, argue that the method of Wong & Collins [3] is inappropriate for examining the tail behaviour of their particular 60 SSEs. The sparsity of data on SSEs is addressed by combining the 15 and 45 observations associated with SARS-CoV and SARS-CoV-2 into a single sample, whereas the two datasets correspond to completely different distributions (figure 2*a*) and should not be pooled accordingly. This is apparent from either a Kolmogorov–Smirnov test, with *p*-value 0.015, or the more common approach making the questionable assumption that *Z* follows a negative binomial distribution. The conditional (given *Z* > 6) negative binomial fit of the probability mass function to the *Z*_*i*_ (by construction larger than 6), calculated as described in the last paragraph of the Methods section (figure 2*b*), already suggests that the upper tail of *Z* for SARS-CoV appreciably dominates that for SARS-CoV-2. In other words, even a naive analysis of the SSE distributions, using the classical negative binomial distribution and not accounting for the heavy tail in the data, indicates that the SSEs for SARS-CoV and those for SARS-CoV-2 exhibit different statistical behaviour. This is confirmed by an analysis of the data properly taking into account its extremes (figure 2*c*): the *ξ* estimates obtained from the Hill estimator in the special case of SARS-CoV-2 vary between 0.35 and 0.45, and as such differ substantially from the various competing estimates found to vary between 0.5 and 1 in [3]. Even the 90% confidence intervals of *ξ* for SARS-CoV-2 (dashed red lines in figure 2*c*) only partially contain the estimated extreme value index plot for SARS-CoV (solid blue line), reflecting a net difference between the two fat-tailed distributions of secondary cases associated with SARS-CoV and SARS-CoV-2. This conclusion is corroborated by the mean excess function estimates (figure 2*d*), which similarly indicate the relevance of separating the analysis for each coronavirus. This suggests that although SARS-CoV and SARS-CoV-2 belong to the same family of respiratory diseases, SSEs are larger in scale for SARS-CoV in comparison with SARS-CoV-2. For all these reasons, pooling the data before applying extreme value tools can lead to misleading conclusions on the propagation of the SARS-CoV-2 virus.

**Figure 3.**
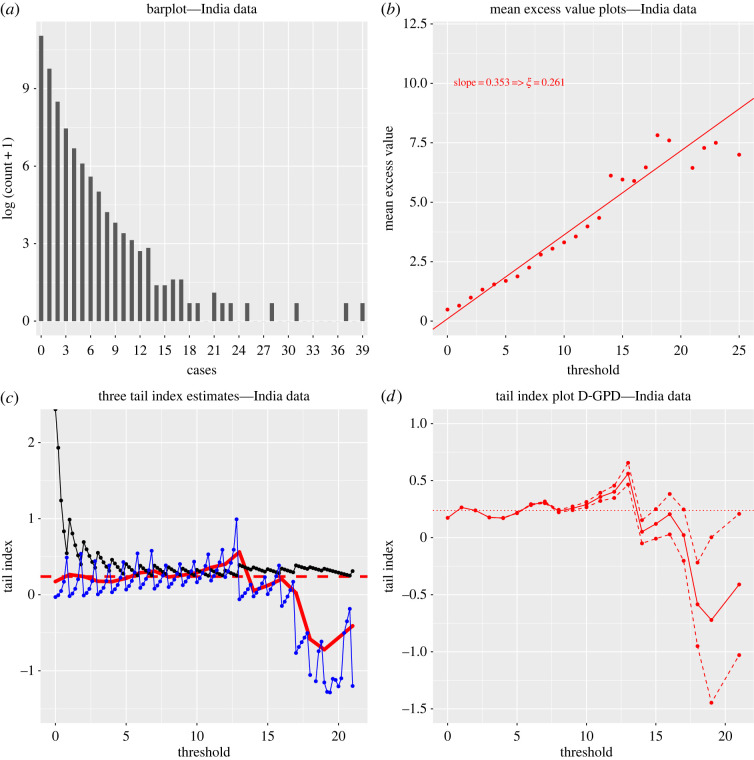
Secondary case data (Database S3) for SARS-CoV-2 from Andhra Pradesh and Tamil Nadu (India). (*a*) Barplot of the log (*Z*_*i*_ + 1) (*n* = 88 527). (*b*) Mean excess plots of secondary cases. (*c*) Hill (solid black), continuous GPD maximum likelihood (solid blue) and discrete GPD maximum likelihood (solid bold red) estimates of *ξ*. (*d*) Discrete GPD maximum likelihood estimates of *ξ* (solid red) and their associated 90% confidence intervals (dashed red). In (*c*) and (*d*), the averaged discrete GPD estimate $\hat{\xi }=0.239$ over the stable region *u* ∈ [0, 10] is indicated with the horizontal red line.

Yet, the low sample size of this SSE dataset puts a question mark over the quality of the statistical analysis. Trustworthy extreme value inference may require a larger sample size, of the order of at least several thousands. This is why we also analysed a much larger Indian secondary case dataset of size *n* = 88 527 (Database S3). This comprehensive surveillance and contact-tracing database was collected in 2020 by the public health authorities of the two Indian states of Andhra Pradesh and Tamil Nadu, whose residents total about 10% of India’s population. It was studied for instance in [4,22], and we refer to the latter for more information about the database’s construction and contents. Results are reported in figure 3. Although the barplot of this data (figure 3*a*) gives evidence of a considerable right skewness and its summary extreme value analysis (figure 3*b*) suggests a heavy right tail, it should be noted that since the *Z*_*i*_ range from 0 to 39 with a sample size of 88 527, the data are necessarily highly discrete with a large number of tied observations (see table 1).

**Table 1.** Secondary case data (Database S3) for SARS-CoV-2 from Andhra Pradesh and Tamil Nadu (India).

*Z*	0	1	2	3	4	5	6	7	8	9	10	11	12	13	14	15	16	17	18	19	21	22	23	25	28	31	37	39
count	62 540	17 493	4885	1730	802	444	267	149	67	44	29	22	14	16	3	3	4	4	1	1	2	1	1	1	1	1	1	1

Ignoring the discrete nature of the *Z*_*i*_ by modelling their tail behaviour with the (generalized) Pareto distribution is inappropriate, as this typically results in unreliable extreme value index estimates and confidence intervals [13]. This becomes obvious here by superimposing both the classical Hill and continuous generalized Pareto maximum likelihood estimators of the extreme value index as functions of a varying threshold *u* in figure 3*c*. Clearly, both plots are so volatile and jagged that it is hard to identify any stable region, and therefore a reasonable point estimate of *ξ* cannot easily be determined. Using the D-GPD distribution to fit exceedances *Z*_*i*_ − *u* above the threshold *u* (rather than trying to fit the whole of the distribution, as Kremer *et al.* [22] did using a discrete Pareto distribution) results in a much smoother and stable fit (figure 3*c*), and leads to an estimate of *ξ* around 0.24 with the 90% confidence intervals overwhelmingly suggesting an estimate greater than 0, thus confirming the fat-tailed nature of SARS-CoV-2 SSEs (figure 3*d*) in this sample. Interestingly, revisiting the small SARS-CoV-2 SSE dataset (Dataset S2) of size 45 using the D-GPD maximum likelihood estimation method (figure 2*e*) results in an estimate of around 0.25, in agreement with the results from the Indian secondary case data. This suggests that the distribution of SARS-CoV-2 SSEs has a finite third moment and possibly even a fourth moment. These results are different from those obtained for the SARS-CoV SSEs. The latter rather point towards a distribution with infinite variance and thus a much heavier right tail. This is confirmed by considering the fitted D-GPD probability mass functions for secondary cases (figure 2*f*) that decrease much more rapidly for SARS-CoV-2 than for SARS-CoV.

To examine the extreme value behaviour of the SARS-CoV-2 offspring distribution in different conditions, we turn to the analysis of two contact-tracing datasets in South Korea, a country which has a similar population density to the Indian state of Tamil Nadu, but did not resort to any full lockdown and has one of the largest and best-organized epidemic control programmes in the world. The first dataset was collected in the first half of 2020 (Database S4), while the second was collected during the fourth community epidemic in the summer of 2021 (Database S5) in the context of the assessment of transmission dynamics for the Delta variant of SARS-CoV-2. The first dataset, which consists of *n* = 5165 numbers of SARS-CoV-2 secondary cases *Z*_*i*_, was analysed in [3] (see table 2).

**Table 2.** Secondary case data (Database S4) for SARS-CoV-2 collected in South Korea in the first half of 2020.

*Z*	0	1	2	3	4	5	6	7	8	9	10	11	12	15	17	18	21	24	27	51
count	4558	364	114	62	27	7	7	4	4	1	2	3	1	2	2	1	2	2	1	1

**Figure 4.**
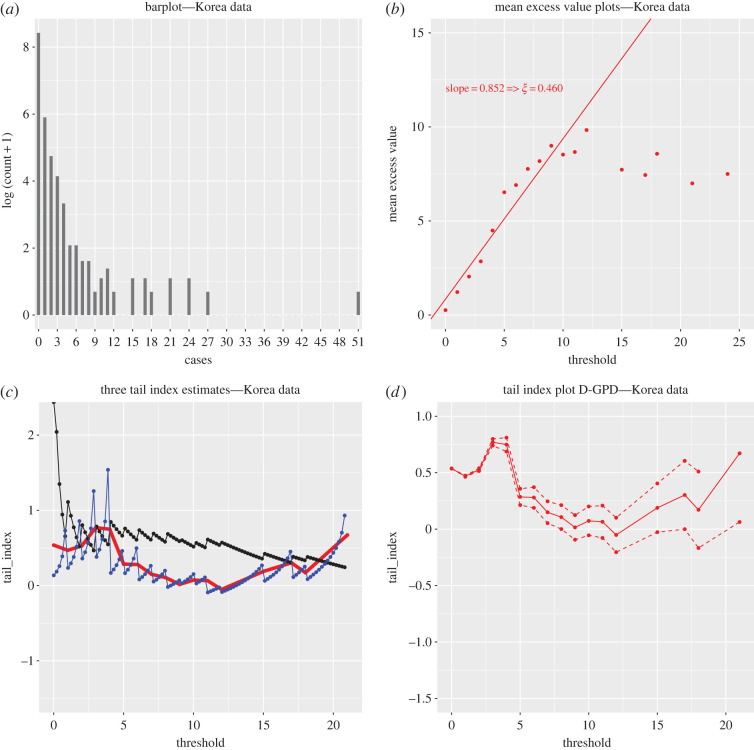
Secondary case data (Database S4) for SARS-CoV-2 from South Korea (first half of 2020). (*a*) Barplot of the log (*Z*_*i*_ + 1) (*n* = 5165). (*b*) Mean excess plots of secondary cases. (*c*) Hill (solid black), continuous GPD maximum likelihood (solid blue) and discrete GPD maximum likelihood (solid bold red) estimates of *ξ*. (*d*) Discrete GPD maximum likelihood estimates of *ξ* (solid red) and their associated 90% confidence intervals (dashed red).

We revisit the estimation of, and inference about, the underlying extreme value index by comparing the D-GPD estimates with the classical GPD and Hill estimates. Results are displayed in figure 4. A least-squares fit to the first part of the mean excess plot (figure 4*b*) suggests a linearly increasing fit to the mean excess function with a slope of around 0.85, but this ignores the flat or even slightly linearly decreasing right-hand part of the data cloud. This throws the assumption that the offspring distribution is fat-tailed in doubt, although the barplot of the data (figure 4*a*) would tentatively back the heavy tail assumption. The Hill estimator, which presupposes that the data are fat-tailed and graphed as a black line in figure 4*c*, does not exhibit any stable region which would allow to produce a reasonable point estimate. In such scenarios, best practice in extreme value theory requires calculating alternative extreme value estimators whose consistency does not rest upon the heavy tail assumption (unlike the Hill estimator), such as the general GPD and D-GPD estimators. These are also represented in figure 4*c*. Clearly, the paths of these two estimates follow a similar trajectory which is very different from that of the Hill plot. They point towards substantially lower estimates of *ξ*, and even though the estimates are overall larger than 0, the validity of the heavy tail assumption *ξ* > 0 is not obvious for this dataset. Figure 4*d* further supports this observation: in the (somewhat) stable region around the threshold *u* = 10, the 90% confidence interval produced through maximum likelihood theory contains the value 0. Our conclusion from the analysis of this dataset is that the distribution of the number of secondary cases is either fat-tailed but with a low extreme value index, or perhaps even has an exponential-type tail. As a consequence, our finding is qualitatively different from that of Wong & Collins [3], since we do not obtain *ξ* estimates similar to those found by merging Datasets S1 and S2.

**Figure 5.**
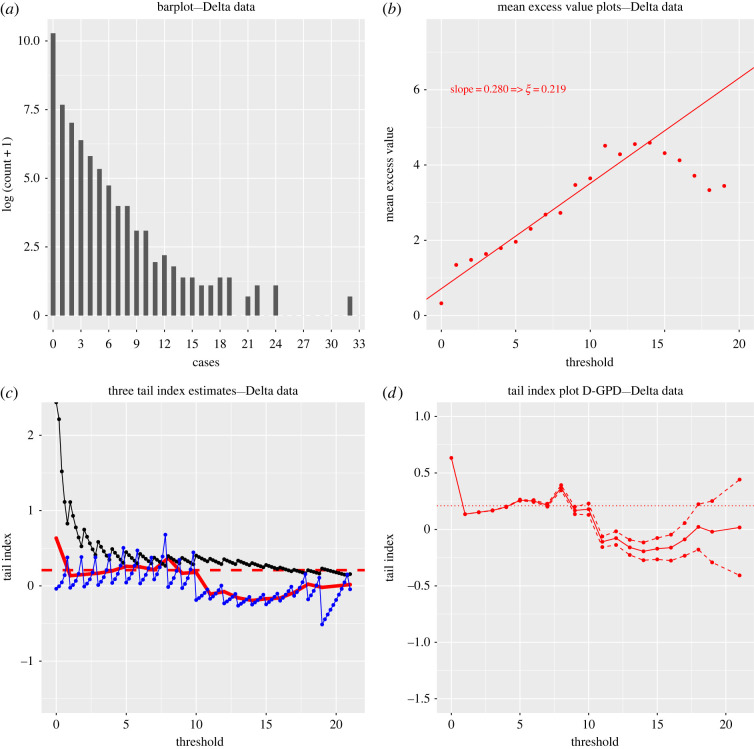
Secondary case data (Database S5) for SARS-CoV-2 from South Korea (July–August 2021). (*a*) Barplot of the log (*Z*_*i*_ + 1) (*n* = 33 903). (*b*) Mean excess plots of secondary cases. (*c*) Hill (solid black), continuous GPD maximum likelihood (solid blue) and discrete GPD maximum likelihood (solid bold red) estimates of *ξ*. (*d*) Discrete GPD maximum likelihood estimates of *ξ* (solid red) and their associated 90% confidence intervals (dashed red). In (*c*) and (*d*), the averaged discrete GPD estimate $\hat{\xi }=0.209$ over the stable region *u* ∈ [1, 10] is indicated with the horizontal red line.

The second South Korean contact-tracing dataset comprises *n* = 33 903 SARS-CoV-2 numbers of secondary cases *Z*_*i*_ (Database S5) detected between 25 July 2021 and 15 August 2021. It was initially explored in [5], where it was highlighted that the Delta variant accounted for the majority of those cases. We, therefore, investigate this dataset to ascertain whether the tail behaviour of SSEs is substantially different for the Delta variant. The data are presented in table 3. The results we obtain for this dataset are displayed in figure 5. The barplot of the data in figure 5*a* again backs the assumption of a heavy tail, but here, the mean excess plot in figure 5*b* suggests a more convincing linearly increasing fit to the mean excess function with a slope of around 0.3. The Hill estimator and both continuous and discrete GPD maximum likelihood estimators, represented in figure 5*c*, appear to support the fat tail assumption of the offspring distribution which is mainly dominated here by the Delta variant. Once again, the D-GPD estimate has a much smoother and more stable sample path, with a stable zone over *u* ∈ [1, 10] indicating a point estimate of around 0.21. The 90% confidence interval of the D-GPD estimate over that region, provided in figure 5*d*, does not contain 0 and offers further justification of the assumption that the offspring distribution is fat-tailed in this dataset, in contrast to the 2020 South Korea data where the validity of this conclusion is much less clear.

**Table 3.** Secondary case data (Database S5) for SARS-CoV-2 collected in South Korea from 25 July to 15 August 2021.

*Z*	0	1	2	3	4	5	6	7	8	9	10	11	12	13	14	15	16	17	18	19	21	22	24	32
count	29 193	2154	1121	594	332	207	113	53	53	21	21	6	8	5	3	3	2	2	3	3	1	2	2	1

### 3.2. Analysis of cluster size data

We broaden our analysis by examining whether SARS-CoV-2 cluster sizes are fat-tailed. We consider a database of 15 samples of cluster sizes recorded in 11 countries and four US states. We define a cluster as a local outbreak involving a minimum of two cases, including confirmed close contacts with epidemiological linkage observed up to extinction of the outbreak. This differs from the number of secondary cases linked to a single, given index case in an SSE, since the cluster size is now the total number of infected people over the duration of the outbreak. The number of reported clusters per country or state varies from 29 (France) to 4769 (Colorado, USA). The database is constructed from government reports [6–9] (Database S6) and media sources [10] (Database S7). The median cluster sizes were 5 (Database S6) and 33 (Database S7), and the largest clusters had sizes 1761 (Database S6, in a Colorado prison) and 7000 (Database S7, in an Italian football stadium). We denote by *Y*_*i*_ the number of SARS-CoV-2 cases in cluster *i*. The *ξ* estimates from each sample of cluster sizes allow to infer the risk category of the corresponding country/state in terms of local community transmission.

**Figure 6.**
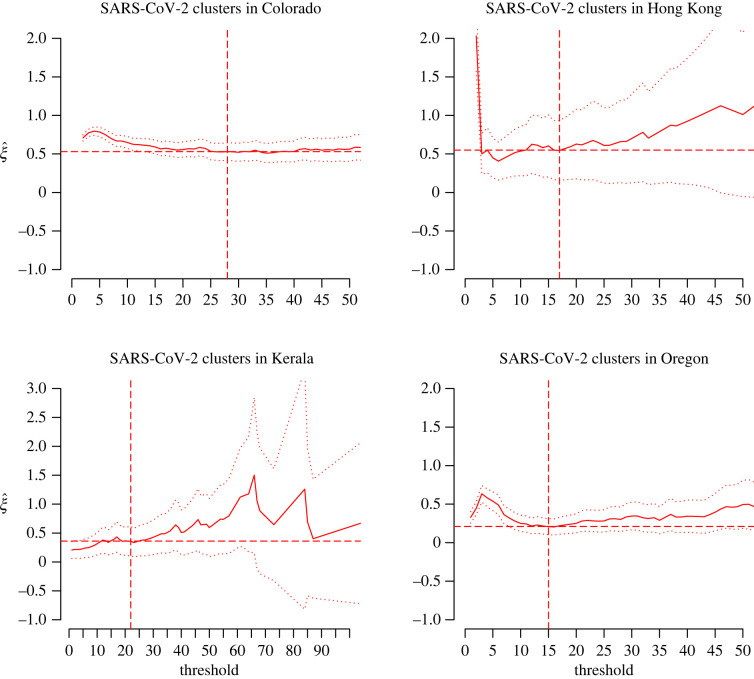
Analysis of cluster cases, for the four countries/states where the source is official data (Database S6). Plots of discrete GPD maximum likelihood estimates of *ξ* (solid lines), along with their 90% confidence intervals (dotted lines) and the final selected estimates (horizontal dashed lines) and thresholds (vertical dashed lines).

**Figure 7.**
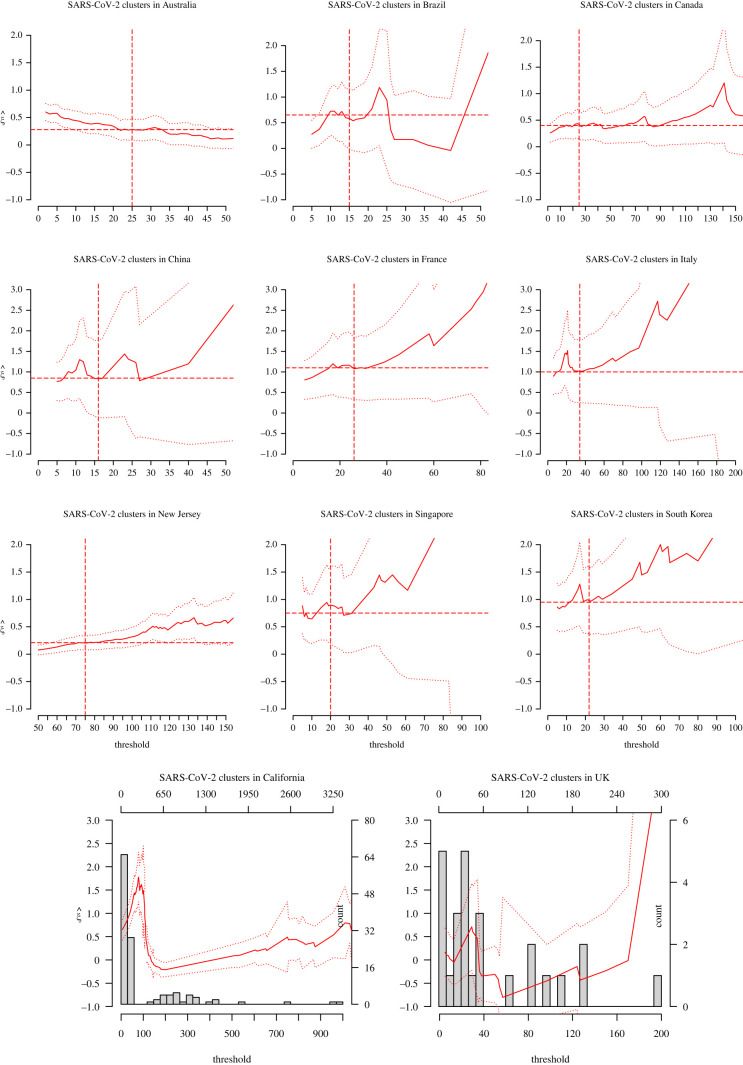
Analysis of cluster cases as in figure 6, with the results obtained from the data whose sources were the media (Database S7). The top nine plots refer to those countries and states for which the extreme value analysis was conclusive. The bottom two plots refer to those for which the extreme value analysis was inconclusive.

Figures 6 and 7 display the D-GPD maximum likelihood estimates of *ξ* as functions of the cluster size *u*. Eyeballed thresholds are indicated by the vertical dashed lines in figures 6 and 7. The final selected estimates are reported in table 4, where 13 out of the 15 countries or states appear to have fat-tailed cluster size distributions (confirmed at the 90% confidence level except for China). We note that there is strong variation in point estimates of *ξ* across countries and states. The low sample sizes of the data available in each case (except for the two US states of Colorado and Oregon) certainly play an important role in that variation. Heterogeneity in population density and healthcare policies may also be substantial factors, although this would have to be cross-checked using complete demographic and public health data. The analysis for California and the UK was inconclusive. For the California dataset, this is possibly due to a strong degree of heterogeneity (see the histogram in the bottom left panel of figure 7). A stratified study of the Californian data might be more conclusive. For the UK dataset, the fact that the sample is so small (26 clusters) in a country with a highly developed healthcare and contact-tracing system is suspicious and may suggest reporting issues.

**Table 4.** Final results for SARS-CoV-2 cluster sizes by country (first column), the corresponding sample size *n* (second column), D-GPD maximum likelihood *ξ* estimate and 90% confidence interval (third column), selected cluster size threshold *u* and associated number *n*_*u*_ of exceedance values *Y*_*i*_ − *u* given *Y*_*i*_ ≥ *u* upon which the *ξ* estimate is calculated (fourth column), D-GPD maximum likelihood 95% and 99% percentile estimates of cluster size (fifth and sixth columns), and the sample maximum (last column). The top table corresponds to data from official sources (Database S6), and the bottom table to data from media sources (Database S7). The results reported in the latter table only concern the nine countries and states for which the extreme value analysis was conclusive.

**Database S6**						
location	*n*	$\hat{\xi }$ [90% CI]	*u* (*n*_*u*_)	${\hat{q}}_{0.95}$	${\hat{q}}_{0.99}$	Max. *Y*_*i*_ (setting)
Colorado, USA	4769	0.53 [0.41, 0.64]	27 (474)	48	140	1761 (prison)
Hong Kong	54	0.55 [0.16, 0.93]	17 (34)	119	310	732 (dancing)
Kerala, India	113	0.36 [0.11, 0.62]	22 (60)	120	255	580 (unknown)
Oregon, USA	795	0.21 [0.10, 0.31]	15 (254)	64	124	639 (prison)

**Database S7**						
location	*n*	$\hat{\xi }$ [90% CI]	*u* (*n*_*u*_)	${\hat{q}}_{0.95}$	${\hat{q}}_{0.99}$	Max. *Y*_*i*_ (setting)
Australia	355	0.28 [0.09, 0.48]	25 (145)	157	326	662 (cruise ship)
Brazil	42	0.58 [0.00, 1.16]	15 (22)	82	220	191 (hospital)
Canada	100	0.42 [0.15, 0.69]	25 (74)	272	624	1500 (meat processing plant)
China	34	0.84 [−0.12, 1.80]	16 (10)	99	401	368 (market)
France	29	1.08 [0.32, 1.83]	26 (17)	443	2530	2500 (religious gathering)
Italy	41	1.02 [0.25, 1.79]	34 (15)	378	2013	7000 (stadium)
New Jersey, USA	183	0.20 [0.08, 0.33]	75 (157)	299	496	1042 (prison)
Singapore	45	0.90 [0.19, 1.61]	20 (21)	156	661	797 (worker housing)
South Korea	45	0.98 [0.37, 1.59]	22 (24)	324	1616	5016 (religious gathering)

Using the D-GPD model, one can gain further insight into large cluster sizes by providing extrapolated estimates of extreme percentiles *q*_*α*_ potentially beyond the sample maximum, through the estimate ${\hat{q}}_{\alpha }$ described in the Methods section. Estimated 95th and 99th percentiles are given in table 4. One may also match the estimated percentiles with actual observations to get a sense of what would constitute a conducive environment for the formation of large SARS-CoV-2 clusters. For example, the estimated 95th percentile of 120 cases in Kerala is close to two clusters of 113 cases (nursing home) and 132 cases (local transmission) already observed in Kerala. Likewise, the estimate ${\hat{q}}_{0.95}=272$ cases in Canada is fairly close to a cluster of 324 cases in Canadian nursing homes. In Oregon, the estimated 99th percentile ${\hat{q}}_{0.99}=124$ cases is in the vicinity of a cluster of 134 cases in a care home setting. In Colorado, the estimate ${\hat{q}}_{0.99}=140$ cases is close to a cluster of 134 cases in a nursing home. All of these clusters bar one (the local transmission cluster in Kerala) correspond to indoor environments where social distancing is difficult to practise.

## 4. Discussion

In summary, we have investigated four datasets of secondary case numbers *Z*_*i*_ for SARS-CoV-2 as a way to estimate and infer the extreme value index of the related underlying offspring distribution. Motivated by the highly discrete nature of such data, we used the discrete GPD (D-GPD) maximum likelihood estimation method, which produces smoother and more stable plots of the associated D-GPD estimator than the classical continuous GPD and Hill estimators. We first provided evidence that the small SSE dataset (Dataset S2) compiled by Wong & Collins [3] during the early phase of the COVID-19 pandemic was fat-tailed, thus confirming their findings, although we show in various ways that this dataset should not be pooled with their 15 SSEs associated with SARS-CoV (Dataset S1), since they correspond to substantially different distributions. On the other hand, as accurate extreme value inference requires a large sample size in general, we also analysed an Indian secondary case dataset of size 88 527 collected in 2020 (Database S3), which contains a very large number of tied observations. The D-GPD estimate of the extreme value index is around 0.24, which is in full agreement with the estimate of around 0.25 found by revisiting the small SSE dataset of size 45 from Wong & Collins [3]. The distribution of SARS-CoV-2 SSEs, therefore, appears to have at least a finite third moment, whereas that of SARS-CoV SSEs is found to have a much heavier upper tail with infinite variance and therefore stronger superspreading effect. In an effort to account for the quality of implemented control programmes as well as the nature of the variant under study, we used two extra South Korean contact-tracing datasets. For the first dataset (Database S4), collected in the first half of 2020 and used in [3], we cannot disprove that the distribution of the number of secondary cases has an exponential-type tail. By contrast, for the second South Korean dataset (Database S5) collected during the summer of 2021, in which the majority of cases correspond to the Delta variant of SARS-CoV-2 [5], we obtained a D-GPD estimate, $\hat{\xi }\approx 0.21$ clearly suggesting a heavier upper tail for the Delta variant and therefore more pronounced superspreading potential in South Korea relative to the first half of 2020.

We broaden our analysis by providing evidence that SARS-CoV-2 cluster sizes are typically fat-tailed, based on 15 samples from 11 countries and four US states. We infer the risk exposure and risk category of each country and state by making use of D-GPD maximum likelihood estimates of both the extreme value index and extreme percentiles, along with their associated confidence intervals. For the sake of simplicity, we used a straightforward threshold selection rule, which is to spot a stability region in the estimates (as a function of the threshold value) and choose an estimate whose value is representative of those reached in this region. This practice, colloquially known as ‘eyeballing’, is standard in applied extreme value analysis: see for example the discussion on p. 77 of chapter 4 in [26]. It applies reasonably well to the D-GPD sample paths, because they are overall much smoother and more stable than the standard Hill and GPD maximum likelihood sample paths, which are not designed to handle the discreteness of the data. The development of more elaborate statistical techniques for the choice of threshold in discrete GPD maximum likelihood estimation, such as methods based on asymptotic m.s.e. minimization or the bootstrap in the spirit of the approaches outlined in section 5.4 of Gomes & Guillou [27] for Hill estimation, is an open question which is beyond the scope of this paper.

**Figure 8.**
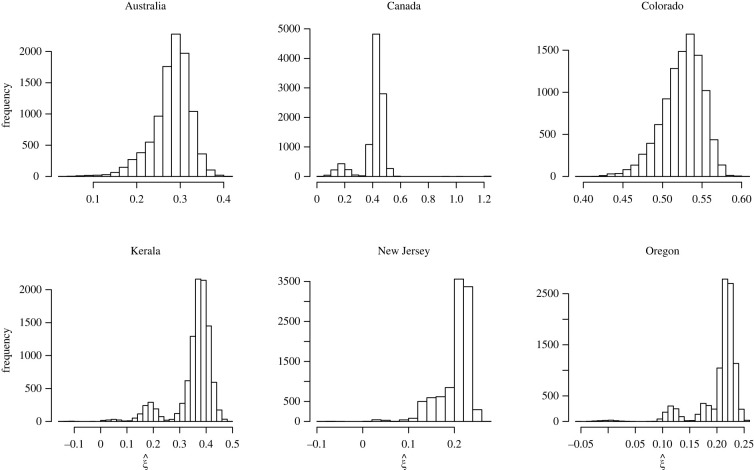
Robustness check (with respect to data omission) for the analysis of cluster cases (Databases S6 and S7). Histograms of the 10 000 estimates of *ξ* obtained by omitting at random 10% of the data. This was done only for the six samples containing at least 100 data points.

**Figure 9.**
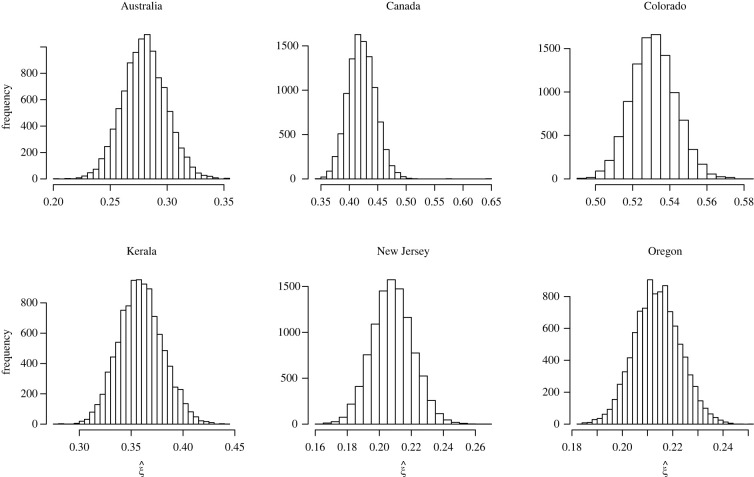
Robustness check (with respect to poor recording of the data) for the analysis of cluster cases (Databases S6 and S7). Histograms of the 10 000 estimates of *ξ* obtained by multiplying each data point by a random draw from the normal distribution with mean *μ* = 1 and standard deviation *σ* = 0.05. This was done only for the six samples containing at least 100 data points.

A limitation of our study lies in the quality of the data, as it is not obvious whether all SSEs or clusters over a given time period were available, or whether cluster sizes were correctly recorded. To check robustness against missing data, we have reproduced part of our analysis of cluster data by removing 10% of observations at random in each sample containing at least 100 data points, and replicating this experiment 10 000 times. Robustness against poor recording was checked by multiplying each observation *Y*_*i*_ by an independent normal variate *W*_*i*_ having mean *μ* = 1 and standard deviation *σ* = 0.05, and then reproducing our analysis of cluster data on the *Y*′_*i*_ = *W*_*i*_ *Y*_*i*_, this experiment being again replicated 10 000 times. There is indeed some variation in the resulting estimates of *ξ* (figures 8 and 9), but this does not affect our conclusion on the fat-tailed behaviour of the data, except in rare situations when almost all the large values in the data go missing. This highlights the importance of accurate data reporting as a prerequisite to such analyses. A further limitation lies in the assumption of independent data that is implicitly made in order to derive confidence intervals for extreme value parameters, even though the data are implicitly time series. Handling serial dependence in the current setting of discrete epidemiological data is obviously an interesting but very difficult question, involving the hitherto open problem of extreme value dependence in discrete time series, which deserves a study of its own.

It should be noted that, in classical epidemiological models, accurate estimation of the basic reproduction number *R*_0_ is of crucial importance, as it informs the extent of restrictions on social interactions and other control measures that should be imposed to terminate the spread of an epidemic. The range of *R*_0_ for SARS-CoV-2 has been revised in [28] to 4.7–11.4, which is considerably higher than most early estimates. This might explain why moderate restrictions that were implemented in some nations, e.g. France, Italy, Spain, the UK, Australia and New Zealand, turned out to be insufficient and replaced by nationwide or statewide lockdowns and/or border closures. It should be clear that our results are, by construction, robust to mis-specified estimates of the expected number of secondary cases *R*_0_ since they solely rely on extreme values of numbers of secondary cases.

Our approach can be viewed as a proof of concept that transmission data from a respiratory disease should not be pooled with data from a similar disease, since similar *R*_0_ numbers or parameters of average transmission do not, in general, induce similar parameters of large community transmission. As such, preparing proactive control measures actually requires a fine assessment of how unequal the distributions of SSEs associated with different SARS-CoV-2 variants are. Liu & Rocklöv [29] conclude that the reproductive number of the Delta variant is far higher than that of the historical SARS-CoV-2 virus. Similarly, Ito *et al.* [30] estimate that the effective reproduction number of the Omicron variant is more than three times that of the Delta variant in Denmark. Our analysis of secondary case data did not, strictly speaking, allow one to conclude statistically that SSEs linked to the Delta variant had a different extreme value index from those linked to the original strains of SARS-CoV-2. However, in the contact-tracing data recorded in South Korea, we did find a heavy tail in the offspring distribution when the Delta variant made the majority of cases, as opposed to when it did not. This tentative finding of a heavier tail in the data linked to the Delta variant is coherent with the higher reproductive number of the Delta variant found in [29]. The question of estimating parameters of large community transmission for the Omicron variant remains open, as we could not find a dataset whose sample size would enable us to draw statistically principled conclusions about the tail behaviour of Omicron-related SSEs.

## Data Availability

Data and relevant code for this research work are stored in GitHub: https://github.com/AntoineUC/SARS-CoV-2-codes and have been archived within the Zenodo repository: https://doi.org/10.5281/zenodo.7509725 [31].
